# Supplementary material for: Altered Brain Criticality in Schizophrenia: New Insights From Magnetoencephalography
Source: Front Neural Circuits. 2022 Mar 28;16:630621. doi: 10.3389/fncir.2022.630621 (PMC8995790; doi:10.3389/fncir.2022.630621)
Supplement: Supplementary file 1 [file Data_Sheet_1.docx]

**Supplementary material 1**


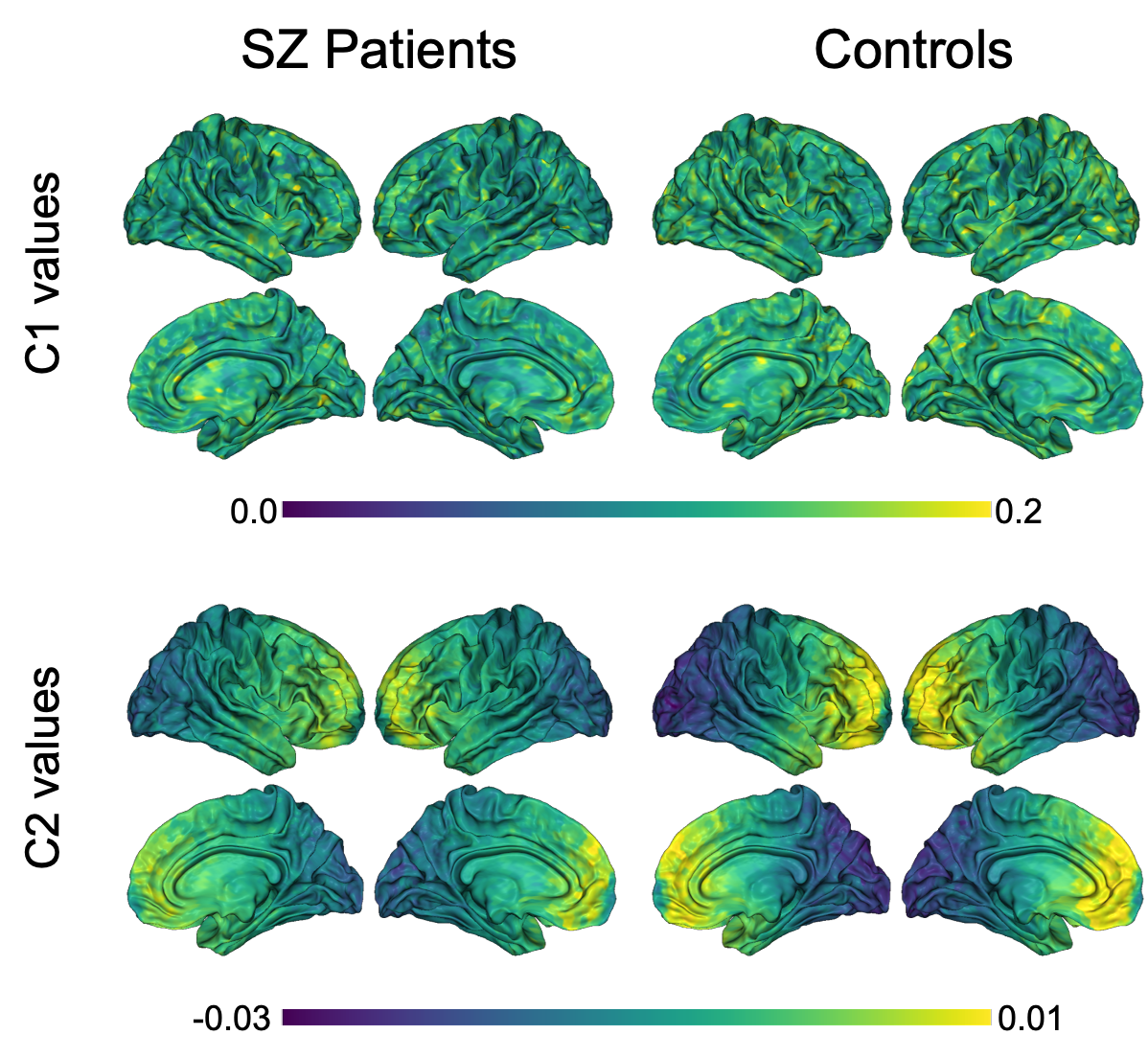


**Supp. Fig.1: Group averages of C1 and C2 values in SZ patients and controls using p=4**. Averaged C1 and C2 values were computed for each of the 8196 nodes, within each group, using p=4. SZ= schizophrenia


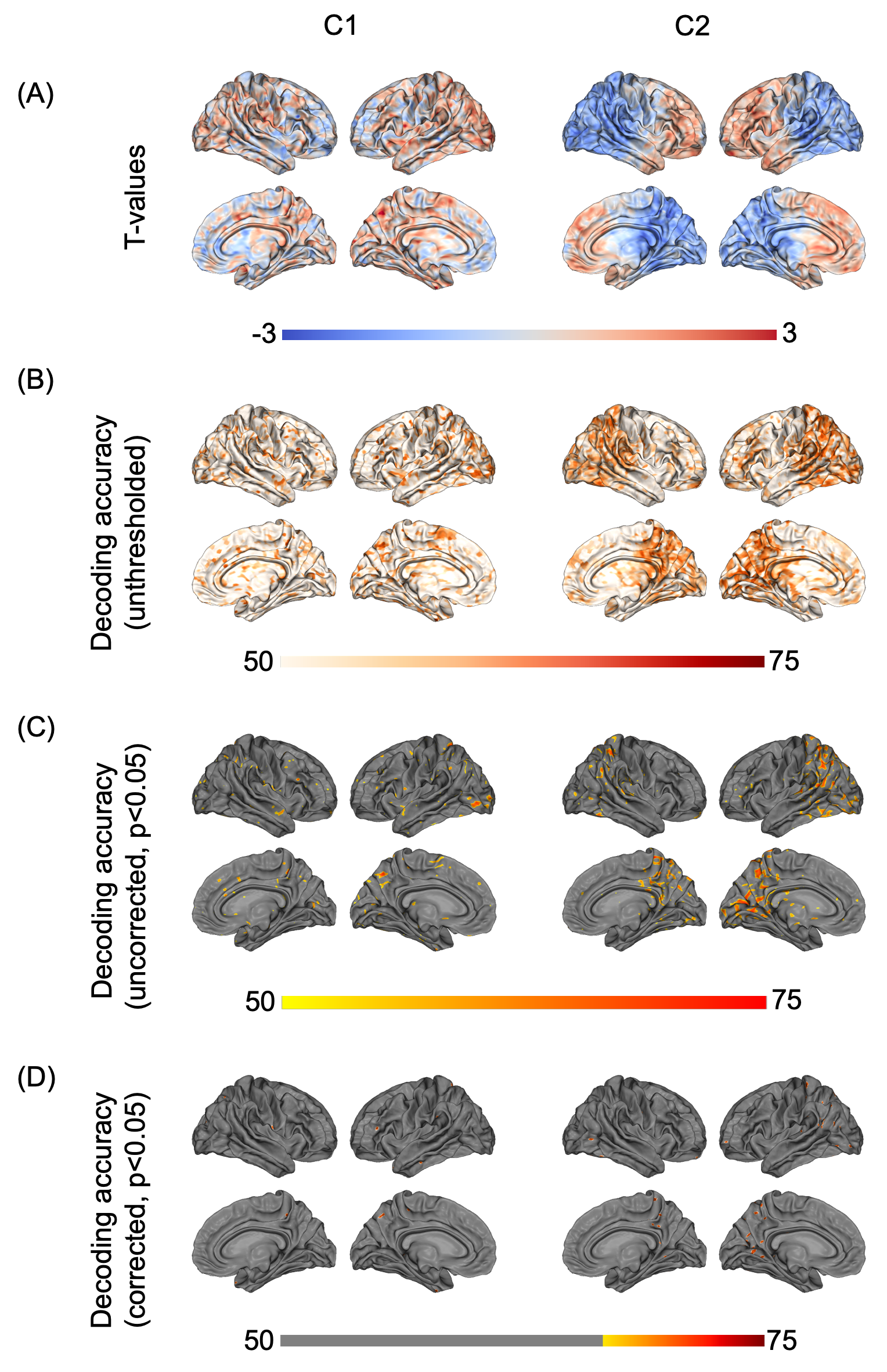


**Supp. Fig. 2: Group differences and machine-learning results using p=4.** (A) shows t-values from the unpaired t-tests (non-significant), showing (controls – patients). (B) shows unthresholded DA values based on logistic regression, using C1/C2 as a single feature. (C) shows the same DA values, thresholded at p<0.05. Finally, (D) shows the DA values corrected for multiple comparisons using maximum statistics (p<0.05), thresholded at the chance level of 70%. P-leader p=4 was used here. DA= decoding accuracy.
